# A conceptual model of cancer care and lived experiences in adolescents and young adults with cancer: results from the multi-national European STRONG AYA project

**DOI:** 10.1007/s00520-026-11170-3

**Published:** 2026-09-25

**Authors:** Nicole Collaço, Samantha Sodergren, Kirsty Way, Urška Košir, Charlotte Cairns, Winette van der Graaf, Olga Husson, Anne-Sophie Darlington

**Affiliations:** 1 School of Health Sciences, University of Southampton, Southampton, S017 1BJ England; 2 Youth Cancer Europe, Cluj-Napoca, Romania; 3 Department of Medical Oncology, Netherlands Cancer Institute, Plesmanlaan 121, 1066 CX Amsterdam, The Netherlands; 4 Department of Medical Oncology, Erasmus MC Cancer Institute, Erasmus University Medical Center, Doctor Molewaterplein 40, 3015 GD Rotterdam, The Netherlands; 5 Department of Public Health, Erasmus MC Cancer Institute, Erasmus University Medical Center, Doctor Molewaterplein 40, 3015 GD Rotterdam, The Netherlands; 6 Department of Surgical Oncology, Erasmus MC Cancer Institute, Erasmus University Medical Center, Doctor Molewaterplein 40, 3015 GD Rotterdam, The Netherlands

**Keywords:** Adolescents and young adults, Cancer care, Conceptual model, Qualitative

## Abstract

**Purpose:**

Adolescents and young adults (AYAs, 15–39 years) with cancer face unique medical, psychosocial and developmental challenges. Existing conceptual models help explain AYA cancer experiences but are often limited by age or national context. The aim of this study was to develop a comprehensive conceptual model reflecting the diversity and complexity of AYA cancer experiences across Europe.

**Methods:**

Within the STRONG AYA project, an exploratory qualitative study was conducted. Fifty-two semi-structured online interviews were completed with AYAs with lived experience of cancer and healthcare, allied health and other professionals involved in AYA cancer care, from European countries. Participants were asked about outcomes most important to AYAs. Data were analysed thematically, and the resulting themes informed a conceptual model.

**Results:**

Four themes were identified: (1) healthcare delivery, access, quality of care and navigation, (2) economic instability and hardship in the cost of survival, (3) fertility and family planning: inequalities in counselling and preservation access and (4) liminal survivorship and societal reintegration. The model encompasses the following cross-cutting domains: *structural/contextual factors* (healthcare systems, policies, societal and cultural influences), *mediators* (information access, social support, financial coverage), *processes* (delayed decisions, fragmented care) and *individual AYA outcomes* (mental/physical health, identity, autonomy, education/work trajectories, relationships).

**Conclusions:**

This conceptual model highlights how AYA cancer experiences/outcomes are shaped and influenced by interacting structural, societal, country-level factors, healthcare systems, cultural contexts and policies across settings. This model may be used to further develop and test culturally sensitive AYA-specific assessments/tools and interventions to improve care and support.

**Supplementary Information:**

The online version contains supplementary material available at https://doi.org/10.1007/s00520-026-11170-3.

## Introduction

Adolescents and young adults (AYAs) with cancer, defined as individuals aged 15 to 39 years at diagnosis [1], differ from younger children and older adults in biological, epidemiological and clinical characteristics, presenting distinct challenges for cancer care [1–4]. In 2022, an estimated 1,300,196 new AYA cancer cases were reported globally, with an annual incidence of 40.3 per 100,000 individuals [5]. Beyond medical challenges, AYAs face age-specific challenges, including treatment late effects, infertility, financial burden and psychosocial concerns that affect education, career progression, romantic relationships and family planning [1, 3, 4, 6, 7]. Despite these challenges, many AYAs report positive changes, such as strengthened relationships, personal growth and greater appreciation for life [3, 7, 8].

Recognising the diverse and evolving experiences of AYAs, healthcare professionals (HCPs) and researchers increasingly seek to understand the unique psychosocial factors shaping the cancer trajectory. However, translating this knowledge into consistent, age-appropriate care remains difficult due to diverse individual needs, limited standardised frameworks and difficulties integrating new evidence into existing care models [9]. Cultural contexts, healthcare systems and societal support structures vary across countries, meaning that two individuals with similar clinical characteristics may experience care differently, potentially leading to divergent outcomes. Globally, AYAs often report inequalities in care [2, 3, 10] and feelings of inadequate support within healthcare systems.

Conceptual models offer one approach to understanding the AYA cancer experience by organising relationships among patient, provider, system and community-level factors that influence cancer experience and care delivery [11].

### Pre-existing conceptual models of the cancer experience for AYAs

Existing conceptual models have largely focused on paediatric populations or older adults [11, 12], or addressed isolated AYA-specific issues such as fertility [13], career development [14] or financial burden [15]. While informative, these models do not capture the full breadth of AYA experiences [11] across the 15–39 age range, or across diverse healthcare contexts.

Two influential conceptual models have characterised the cancer experience [16] and survivorship care of AYAs [11]. Fern et al. [16] developed an AYA-specific model outlining how *quality of life* (QoL) for AYAs is influenced by interconnected mediators, including *symptom experience, information, communication, social support, financial impact* and *ability to cope,* with the *experience of healthcare* shaping these mediators. A key transition is the *end of treatment*, when AYAs *re-establish identity* and work toward *returning to normality* and *achieving life goals*. Gray et al. [11] proposed a patient-centred, cyclical survivorship model placing AYAs and their support network at its core, incorporating domains such as care coordination, mental health, peer support, fertility preservation, financial burden, QoL and education about side effects and late effects.

Although both models have advanced understanding of AYA cancer experiences, their applicability is limited by context and population. Fern et al.’s model [16] was developed in a UK cohort aged 13–25 years and adopts a largely linear structure, while Gray et al.’s [11] model was derived from a relatively homogenous, older survivorship sample recruited primarily through online support groups. Further work is needed to encompass diversity, evolving needs and cross-cultural complexity across the full AYA age spectrum and different healthcare contexts.

Recognising these limitations and the need for a more comprehensive, culturally adapted model (i.e., not embedded within one cultural context), this study integrates the findings from the STRONG AYA project, a European initiative aimed at understanding AYA cancer care needs [10]. Conducted as part of a work package to develop a Core Outcome Set for AYAs with cancer [10, 17–19], this study synthesises these data into a conceptual model that illustrates how clinical, psychosocial, contextual and organisational factors interact to shape the cancer experiences. It can subsequently be used to plan age-specific care, improve the selection of outcomes that reflect AYA priorities in research and inform service planning by highlighting gaps and inequities, particularly at key transition points.

## Methods

The Consolidated Criteria for Reporting Qualitative Research (COREQ) guidelines [20] were followed in reporting the study findings (Online Resource 1).

This paper reports an exploratory qualitative study design [21] conducted in two stages with the following aims: (1) to explore the perspectives and experiences of outcomes important to AYAs with lived experience of cancer and healthcare/allied health and other professionals (HCAOPs) involved in AYA cancer and (2) to develop a conceptual model grounded in themes derived from these perspectives.

An exploratory qualitative approach was used to capture the experiences and priorities of AYAs with cancer and the professionals supporting them, allowing for contextualised understanding across diverse healthcare systems.

### Participant recruitment

Inclusion criteria were AYAs with lived experience of cancer (with or without active disease), diagnosed between 15 and 39 years and HCAOPs with expertise in AYA cancer. All participants were required to be proficient in English and able to provide informed consent. AYAs receiving palliative or hospice care were not eligible.

Participants were recruited using a convenience sampling approach; AYAs were recruited through Youth Cancer Europe via email and social media posts (LinkedIn, Facebook, Instagram). HCAOPs were invited through (inter)national AYA oncology networks by email or newsletter. All respondents who expressed interest and met the inclusion criteria were interviewed.

Interested participants received a participant information sheet by email and, following informed consent, an online interview was scheduled. AYAs received a €25 voucher for participation.

### Ethical considerations

Ethical approval was obtained from the University of Southampton Ethics Committee (ERGO 78653).

### Data collection

Interviews were conducted via Microsoft Teams by two experienced female qualitative researchers with a background in cancer care (NC, KW). The researchers had no prior relationship to the study participants and were unknown to the participants. No additional individuals were present during the interviews. Interviews lasted up to 120 min for AYAs and 30 min for HCAOPs. Interviews were semi-structured; informed by a topic guide asking about issues of importance to AYAs with cancer (Online Resource 2). Field notes were written during interviews to support analysis. Interviews were audio-recorded and transcribed using Microsoft Teams’ integrated transcription function, with transcripts reviewed for accuracy by NC. Data were collected between April and June 2023. Reflexive notes were maintained to consider the influence of researcher roles, assumptions and interactions on data collection and interpretation. This included consideration of how researchers’ backgrounds in AYA cancer research, prior knowledge of AYA cancer experiences and familiarity with existing conceptual models may have influenced interpretation of the data and development of the conceptual model.

### Data analysis

The recruitment target was guided by the concept of information power [22], ensuring sufficient depth and relevance to address the research question.

**Fig. 1:**
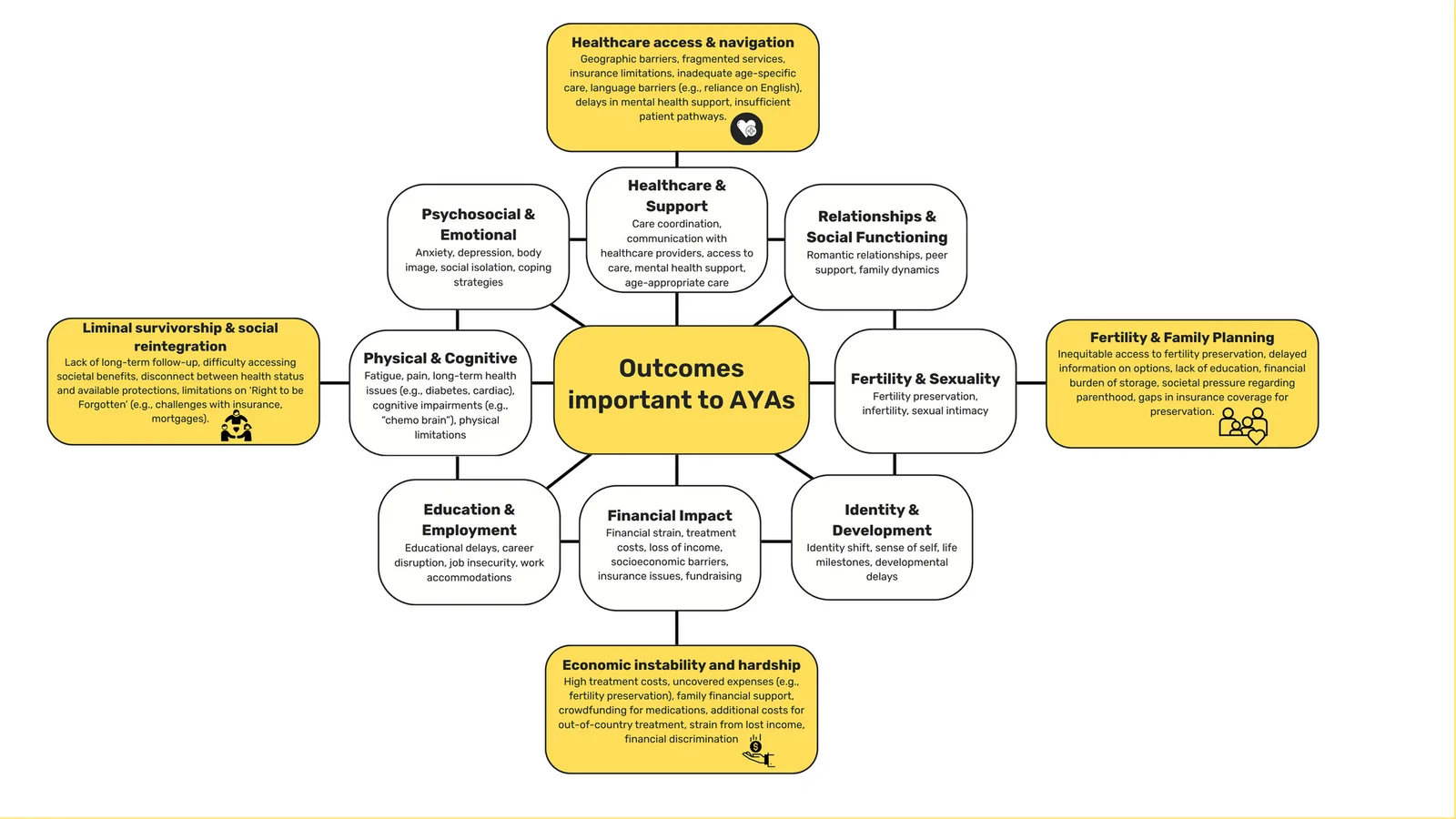
Themes from study findings

Audio recordings were analysed using thematic analysis [23, 24], combining inductive and deductive approaches. Analysis began with data familiarisation through repeated reading of transcripts. Open coding was conducted by the lead researcher (NC) to identify codes related to outcomes important to AYAs. These codes were then organised into broad outcome domains (e.g. education and employment, relationships and social functioning, fertility and sexuality) presented in Fig. 1 and Online Resource 3. These outcome domains were then used as flexible analytic frameworks to organise subsequent analysis while allowing more specific patterns and contextual differences within each domain to be captured.

**Fig. 2:**
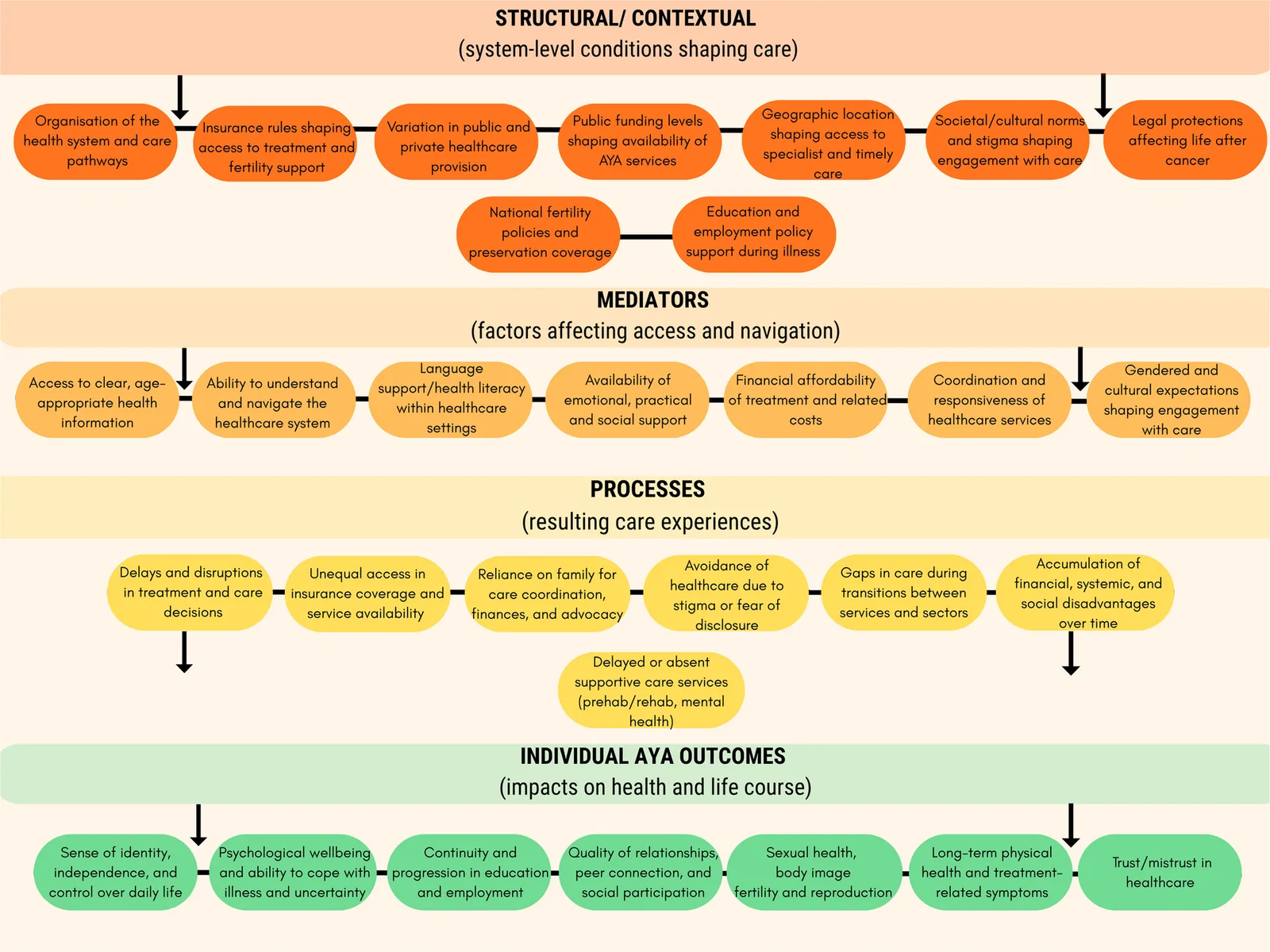
Conceptual model of cancer care and lived experiences in AYAs with cancer. The model illustrates how AYAs with cancer experience care and survivorship across specific European countries. *Structural/contextual* factors are systemic, societal and country-level conditions, healthcare systems, policies, cultural norms and legal protections that shape the cancer care environment. *Mediators*, such as access to information, social support, financial coverage and system responsiveness, shape how structural and contextual factors are experienced by AYAs. *Processes* are the resulting experiences, including delays, fragmented care, reliance on family and missed support. These experiences influence *individual AYA outcomes*, including mental and physical health, identity, relationships, autonomy, education and work and trust in healthcare. The model demonstrates how systemic conditions cascade through mediators and processes to affect AYAs’ lives

The same coding framework was applied to AYAs’ and HCAOPs’ transcripts, reflecting substantial overlap in how outcomes were described by both groups (Online Resource 3). Coding was iterative, and discrepancies were resolved through regular discussions among researchers (NC, CC, KW) to refine the coding framework, ensuring consistency and reliability. A subsequent deductive analysis examined how experiences within each outcome domain varied across countries and contexts (Online Resource 4). This informed the development and refinement of themes (Fig. 1, outer ring) describing similarities and differences in how AYAs experienced these outcomes across settings which informed the final conceptual model (Fig. 2). Microsoft Word and Excel were used for data management and analysis.

## Results

A total of 52 interviews were conducted. Twenty-seven AYA interviews were conducted, of which AYAs (18 female, 9 male) were aged between 23 and 43 years at interview, diagnosed between ages 15 and 36 years, of different nationalities (*n* = 16), and most AYAs were off-treatment (*n* = 24). The mean time since diagnosis was 8.74 years (range, 1–20 years). The 25 HCAOPs (17 female, 8 male) ranged from 28 to 65 years old, from various nationalities (*n* = 12), with experience from 6 months to 34 years in various oncology specialties. Participant characteristics are reported in Table 1.

**Table 1:** Participant characteristics

AYAs									
**Age at interview (range)**	**Age at diagnosis (range)**	**Sex**	**Ethnicity**		**Nationality**		**Diagnosis**		**On/off treatment**
23–43 years 21–30 (*n* = 14) 31–40 (*n* = 12) 41–45 (*n* = 1)	15–36 years 15–20 (*n* = 15) 21–30 (*n* = 8) 31–40 (*n* = 4)	F (*n* = 18) M (*n* = 9)	White (*n* = 25) Latino/Latinx (n = 1) Hispanic (*n* = 1)		British (*n* = 7) Portuguese (*n* = 3) Polish (*n* = 2) Romanian (*n* = 2) Belgian (*n* = 2) Czech (*n* = 1) Croatian (*n* = 1) Dutch (*n* = 1) Dutch/dual citizenship (*n* = 1) Greek (*n* = 1) Lithuanian (*n* = 1) Slovenian (*n* = 1) Spanish (*n* = 1) Swedish (*n* = 1) Ukrainian (*n* = 1) Armenian (*n* = 1)		Leukaemia (*n* = 7) Hodgkin’s lymphoma (*n* = 8) Non-Hodgkin’s lymphoma (*n* = 2) Sarcoma (*n* = 3) Ovarian cancer (*n* = 3) Breast cancer (*n* = 1) Appendicular cancer (*n* = 1) Pancreatic cancer (*n* = 1) Multiple cancers (*n* = 1)		Off treatment (*n* = 24) On treatment (*n* = 3)
Healthcare/allied health/other professionals									
**Age at interview (range)**	**Sex**	**Nationality**		**Profession**		**Experience**		**Core expertise**	
28–65 years	F (*n* = 17) M (*n* = 8)	British (*n* = 10) Belgian (*n* = 2) Dutch (*n* = 2) Portuguese (*n* = 2) Polish (*n* = 2) Czech (*n* = 1) French (*n* = 1) Italian (*n* = 1) Indian (*n* = 1) Norwegian (*n* = 1) Swedish (*n* = 1) New Zealander/British (*n* = 1)		Oncologist (*n* = 6) Consultant (*n* = 2 Senior consultant (*n* = 1) Surgical oncologist (*n* = 1) Clinical psychologist (*n* = 1) Pediatrician (*n* = 1) Nurse specialist (*n* = 4) Lead nurse (*n* = 3) Nurse practitioner (*n* = 2) AYA co-ordinator (*n* = 1) Medical social worker (*n* = 1) Counsellor (*n* = 1) Researcher (*n* = 1)		6 months–34 years		Teenage and young adult oncology/AYA cancer care (*n* = 9) Oncology (*n* = 4) Haematology oncology (*n* = 2) Pediatric oncology (*n* = 1) Pediatric and adolescents (*n* = 1) Psychology (*n* = 1) Early phase clinical trials (*n* = 1) Early drug development, epidemiology and cancer and preventative oncology (*n* = 1) Leukaemia (*n* = 1) Gastrointestinal oncology (*n* = 1) AYA sarcomas/brain tumours (*n* = 1) AYA bone and soft tissue sarcoma (*n* = 1) AYA breast, testicular and neuroendocrine cancer (*n* = 1)	

Four main themes informed the conceptual model of AYAs experiences (Fig. 1): (1) healthcare delivery, access, quality of care and navigation; (2) economic instability and hardship in the cost of survival; (3) fertility and family planning: inequalities in counselling and preservation access and (4) liminal survivorship and societal reintegration.

### Outcomes important to AYAs

The following outcomes were identified as most important to AYAs (also reflecting the HCAOPs’ perspective) (Online Resource 3; Fig. 1), including fertility concerns, relationships and intimacy, psychological, identity and self-perception, social functioning, financial implications, late effects and healthcare experiences. Both positive and negative experiences were reported with variations evidenced across countries. Within the conceptual model (Fig. 2; Table 2), these outcomes sit at the individual level and are influenced by the broader themes described above. We structure the following four themes according to structural/contextual, mediators, processes and individual AYA outcomes.

**Table 2:** Structural/contextual, mediators, processes, and individual factors shaping AYA cancer experiences

Domain	Meaning
Structural/contextual ***(macro-level factors (societal and country level factors, healthcare systems, policies, cultural norms, legalities) that create the environment in which AYAs experience cancer care)***	**Healthcare system design and organisation** • Structure and availability of age-specific or multidisciplinary care affecting access, timeliness and coordination Example: Some countries have specialised AYA services; others have fragmented adult/pediatric transitions • Shapes the foundation for healthcare access and mediates subsequent experiences
	**Insurance regulations and coverage** • Public and private insurance regulations affecting what treatments, tests or services are covered Example: Some AYAs faced gaps in coverage for tests or fertility preservation, creating financial barriers • Influences financial burden and ability to access timely treatment/care
	**Public/private healthcare** • Interaction between public and private healthcare systems that can introduce delays or inequities Example: Navigating referrals between private clinics and public hospitals caused fragmented care and disrupted follow-up • Impacts continuity of care and timeliness of treatment
	**Funding** • Adequate resourcing of hospitals, AYA programmes, and psychological services Example: Underfunded mental health services led to long waiting lists and reduced access • Affects healthcare system responsiveness and availability of support
	**Geography and regional inequality** • Distance from treatment centres and regional resource disparities affecting access Example: AYAs in remote areas had to travel long distances for scans or specialist appointments • Influences practical and financial burdens as well as access to timely care
	**Cultural norms, stigma and taboos** • Societal expectations around gender, health and fertility shaping attitudes and behaviours Example: Some young men tended to avoid psychological care due to norms of masculinity. Cultural or societal taboos also made it hard for young people to talk openly about infertility, and many carefully chose what to share about their cancer with partners to fit social expectations about health, relationships, and family • Influences help-seeking behaviour and engagement with services
	**Legal protections** • Laws safeguarding post-treatment rights, e.g. ‘Right to be Forgotten’ for insurance or mortgages • Example: Lack of protections restricted some AYAs’ ability to access mortgages or other financial loans • Impacts economic stability and societal reintegration
	**Fertility policies and coverage** • Regulations and funding for fertility preservation options Example: Male AYAs often had quicker access to sperm banking than females needing egg harvesting • Influences fertility-related decision-making and long-term life planning
	**Education and employment policies/supports** • Policies/institutional rules that allow AYAs to continue school, university, or work during treatment and in the recovery period Example: Limited support for returning to school or work delayed reintegration • Affects autonomy, independence, and long-term socio-economic outcomes
Mediators ***(factors that shape how AYAs experience these structural conditions, influencing how care and support are accessed and understood)***	**Access to clear, age-appropriate information** • Availability of understandable, age relevant information about diagnosis, treatment, fertility, and long-term effects Example: AYAs received paternalistic explanations or discovered fertility options by accident • Influences decision-making, treatment adherence, and life planning
	**Health literacy and understanding of the healthcare system** • Ability to navigate complex systems and interpret treatment and support options Example: Confusion about pediatric vs. adult protocols influenced engagement with care and sense of control • Shapes comprehension, confidence and effective use of services
	**Language access and communication support** • Availability of consultations and written information in a language the AYA understands Example: Without English-language access, one participant would have been *‘totally in the dark.’* • Directly influences informed consent, treatment understanding and awareness of available services
	**Social and family support** • Emotional, practical, and logistical support from parents, partners or peers Example: Family helping with appointments, childcare or finances; peer support during hospital stays • Buffers the impact of fragmented services, financial burden, and emotional strain
	**Mental health and psychological support availability** • Access to psychologists, support groups and ongoing emotional care Example: Long waiting lists or distant services, especially for young men reluctant to seek help • Mediates stress, coping and emotional outcomes
	**Financial coverage and practical support** • Insurance, out-of-pocket costs and public funding for treatment, medications, fertility preservation, travel and childcare Example: Crowdfunding medications, paying for fertility storage or difficulty returning to work • Mediates economic stability, autonomy and reintegration
	**System responsiveness and coordination** • Efficiency and organisation of care, including wait times, multidisciplinary teams and smooth transitions between different parts of the healthcare system (e.g. pediatric to adult services, hospital to community care or private to public services) Example: Delays in scans or referrals; fragmented follow-up care • Influences timeliness, continuity of care and satisfaction of care
	**Cultural and societal expectations influencing help-seeking** • Norms around masculinity, stigma or shame influencing willingness to seek care or psychological support Example: Perceived expectations to ‘bluster through treatment’ influenced whether AYAs sought emotional support • Mediates emotional wellbeing and engagement with services
Processes ***(the actual experiences of AYAs as they navigate care, shaped by both structural/contextual factors and mediators)***	**Delayed or disrupted decision-making** • Waiting times, misdiagnosis or limited guidance slowing treatment or care decisions Example: Misdiagnosis delaying start of chemotherapy • Links mediators (information access, system responsiveness) to individual outcomes by affecting treatment timing, planning and stress
	**Gatekeeping and unequal access** • Differences in insurance, coverage or service availability limiting access Example: Private insurance not covering certain tests, requiring referrals to public hospitals • Contributes to financial strain and fragmented care, and inequities in outcomes
	**Family involvement (system—family)** • Reliance on family for care, finances or navigating healthcare Example: Parents arranging transportation, paying for tests or handling administrative tasks • Links structural constraints to economic and emotional outcomes
	**Avoidance of help-seeking/stigma** • Withholding needs due to cultural norms, gender expectations or shame Example: Young men ‘blustering through treatment’ and avoiding seeking help for emotional or fertility concerns due to perceived stigma • Affects mental health, engagement with services and coping
	**Fragmented transitions** • Gaps when moving between pediatric/adult care or private/public systems Example: Disjointed follow-up care or delayed access to specialists • Creates discontinuity in care and affects emotional and physical outcomes
	**Cumulative disadvantage** • Compounding effects of financial, systemic and social barriers over time Example: Long travel distances, delayed care and financial strain accumulating during treatment • Amplifies risk for poor quality of life outcomes
	**Missed or delayed supportive care** • Gaps in fertility preservation, mental health, rehabilitation or follow-up services Example: Fertility counselling offered too late or mental health support unavailable • Influences long-term health, psychological outcomes and autonomy
Individual AYA outcomes ***(the personal-level impacts resulting from the interplay of structural/contextual factors, mediators, and processes)***	**Identity disruption** • Changes to sense of self due to illness, treatment or loss of independence Example: Feeling defined by cancer or disconnected from peers • Impacts mental health and social reintegration
	**Mental health and emotional strain** • Anxiety, depression, fear of recurrence or PTSD Example: Isolation leading to survivor guilt or social anxiety • Mediated by access to psychological support and social/family networks
	**Autonomy and independence** • Reliance on others for daily tasks or decision-making; delayed life milestones Example: Moving back home or needing help with daily routines • Affects life planning, education, work and social functioning
	**Relationships and social life** • Impacts on friendships, family dynamics, romantic and peer connections Example: Social isolation, disrupted romantic relationships or altered family roles • Linked to cultural norms, stigma and system support
	**Education and work trajectories** • Delays, disruptions or career changes due to illness or treatment Example: Dropping out of school, taking leave from work or career redirection • Influenced by education and employment policies, the responsiveness of healthcare and support systems, and financial assistance, which can either reduce or exacerbate disruptions to school, work or career plans
	**Sexuality and body image** • Effects of treatment on intimacy, sexual function and body confidence Example: Hair loss, infertility or low libido affecting romantic and sexual relationships • Influenced by fertility support and societal expectations
	**Physical symptoms and long-term side effects** • Fatigue, pain, organ-specific issues, cognitive impairment or ongoing health complications Example: Peripheral neuropathy, chronic pain or chemo brain • Impacts daily functioning, autonomy and mental health
	**Trust or mistrust in healthcare** • Satisfaction with care, perceptions of support and confidence in professionals Example: Feeling ignored or not taken seriously by healthcare staff • Influences future engagement with services and adherence to follow-up

#### Healthcare delivery, access, quality of care and navigation

Differences in how healthcare services were organised and delivered across European settings directly impacted AYAs’ access to, navigation of and experience with cancer care at the time of their diagnosis, which for some participants was almost a decade ago. Geographic, financial and systemic barriers often made navigating care challenging. One AYA reported, “We found the surgeon in the other clinical hospital like 200 km away from my hometown…” (AYA035: 24 years at diagnosis, Female). Healthcare systems also varied in their ability to provide coordinated care; some countries offered specialised support through multidisciplinary teams, while others struggled with fragmented services. AYAs and HCAOPs reported significant challenges arising from transitions between private and public healthcare systems. For some, insurance limitations obstructed full coverage leading to referrals to public hospitals where wait times were a problem, “So I went private […], and my insurance wouldn’t cover it” (AYA002: 22 years at diagnosis, Female).

AYAs described frustration with delays and lack of age-appropriate care, especially during the transition from pediatric to adult care. One HCAOP acknowledged this challenge, stating, “The problem with young adults is that they shift from this pediatric care and they are put in the normal system for adults, which have of course have much more patients and much less dedication for such individual patients […]” (HCAOP028: Male, surgical oncologist). Inconsistencies between age-specific services and adult care presented additional complications, especially for AYAs who are often unfamiliar with healthcare systems.

Another salient issue was limited access to information, particularly when language barriers were present and when age-specific information was needed. One AYA reported, “…it’s good that I had access to English language, if I would not know the language, I pretty much would be probably in the dark totally, and only know information in my local language… the only source of information is your haematologist, which again is very paternalistic and doesn’t want to explain things to you” (AYA038: 18 years at diagnosis, Male).

Lack of mental health support was another major gap, with many AYAs unable to access timely psychological care due to underfunding; “… so we’ll have a lot of patients with really significant mental health problems who don’t meet the criteria to be seen or they’ll get put on a waiting list for several years and then eventually they’re just sort of sign posted to online resources” (HCAOP002: consultant oncologist). In some countries, efforts are being made to address this, including the creation of AYA care coordinators and government investments in AYA cancer centres. Despite this, societal perceptions around masculinity compound these issues, with some young men avoiding mental health care and appearing to “bluster their way through treatment and have this I'll be fine approach” (HCAOP002: consultant oncologist). Stigma surrounding health issues and cultural taboos added further stress. In one case, a young woman felt ashamed or uncertain about seeking essential medical care: “I grew up in a really small village, so I had kind of a closed mind about a doctor seeing my vagina…I was ashamed that someone would see, so it took me like quite some time to want to go…” (AYA002: 22 years at diagnosis, Female).

In addition, healthcare systems overwhelmed by demand led to significant delays in accessing certain services. One AYA reported that it took almost 2 years to see a psychiatrist and was then referred to an off-site psychologist, rather than with their affiliated primary treatment centre. This arrangement led to further disruptions in continuity of care. Meanwhile, political and cultural climates also influenced access and attitudes towards healthcare in certain countries, for example additional restrictions on treatments such as medical cannabis for pain management: “[country] is not kind of a country that you can easily get a permission for medical cannabis…” (AYA31: 30 years at diagnosis, Female).

**Table:** 

Healthcare delivery, access, quality of care and navigation (structural/contextual/mediators/processes)
Structural/contextual: national and regional differences in healthcare systems, insurance coverage, service organisation, geography
Mediators: access to age-appropriate information, system responsiveness, social support
Processes: navigating care, delays, fragmented transitions, reliance on family, avoidance of help-seeking
Individual AYA outcomes: increased psychological distress, unmet physical and mental health needs, disrupted education or employment, reduced autonomy, and challenges in social reintegration

#### Economic instability and hardship in the cost of survival

The financial burden of cancer treatment encompassed treatment and medication costs as well as indirect costs, such as loss of employment during treatment, difficulties returning to work and financial discrimination in survivorship. Participants highlighted disparities in healthcare coverage, out-of-pocket expenses and reliance on family financial support or community fundraising to cover healthcare costs. AYAs reported that without savings, many families face significant financial strain, particularly when large upfront payments are required. While some healthcare systems provided considerable support, others had gaps in covering costs for additional medications, specialised post-treatment needs or urgent procedures. One participant described using personal networks and crowdfunding to source expensive, overseas medications; “I actually put it on Facebook because I myself had to have a fundraiser for treatment… the moment I realised I’m not able to continue working” (AYA 32: 36 years at diagnosis, Female). One participant reported the high cost of care (not covered by insurance) relative to their family’s earnings; “… I had to pay for some tests to be done because they were not covered. And these tests were costing like €200, which again is nearly all of the salary… I have saved up some money from the work that I was doing in in the summer, so I was sometimes paying for that. Sometimes my parents were paying… my family was not well off” (AYA 38: 18 years at diagnosis, Male). For many, this financial strain was intensified by disruptions to work or education. A HCAOP noted that in their country, young adults faced challenges in returning to work too soon to avoid financial impacts or risk their health deteriorating further: “The point is that from the moment they are calling in sick, from the moment they start treatment…they feel pressure to not call in sick or they go back too fast” (HCAOP01: Female, nurse practitioner).

Some countries provided more robust support in terms of covering treatment and medication costs, with patients only required to pay a nominal fee for hospital stays and medical visits. However, even in these systems, certain expenses, such as fertility preservation, were not covered and required paying out of pocket. The economic repercussions of treatment extended beyond finances, creating barriers to social reintegration. Employment discrimination and job insecurity hindered some AYAs in returning to work, limiting their ability to regain independence and stability post-treatment.

**Table:** 

Economic instability and hardship (structural/contextual/mediators/processes)
Structural/contextual: healthcare funding, insurance policies, employment protections, societal support
Mediators: financial coverage, access to assistance, family/community support
Processes: crowdfunding, delayed or missed treatment, pressures to return to work early
Individual AYA outcomes: economic hardship, financial stress, delayed or disrupted education and employment, reduced independence, social reintegration challenges, and long-term vulnerability to financial instability

#### Fertility and family planning: inequalities in counselling and preservation access

Inequalities in fertility counselling and preservation highlight another theme identified across the countries represented in the sample. Male AYAs generally had faster and easier access to sperm banking, whereas female AYAs faced longer, more complex procedures that could delay cancer treatment. These disparities often reflect pre-existing biases and create significant challenges, particularly for women: *“*If you’re a male, you tend to get offered sperm banking because it’s a very quick process…you can book you in for the same day… Whereas if you’re a female, it can take between four and eight weeks to harvest your eggs” (HCAOP08: Female, lead nurse).

Many AYAs reported learning about preservation options too late, and in some cases, discovering them by accident. As one participant reported, “I wish I had better information in the beginning because… I only learned about this risk and the option of preserving accidentally, not from a doctor” (AYA032: 36 years at diagnosis, Female). The lack of fertility education and support was echoed by a HCAOP who emphasised the lack of regulation surrounding fertility care post-treatment, and noting that discussions about future fertility should be a part of the ongoing support post-treatment: “And also, to discuss the fertility in the future, what are the possibilities? Does the fertility come back or not? These things are… that is not well regulated at the moment…” (HCAOP018: Female, oncologist).

There is also unequal access to fertility preservation, as some healthcare systems cover the costs of storing eggs or sperm, while others place the financial burden on patients. One participant explained, “I have to pay every two years for the storage of the frozen sperm" (AYA026: 19 years at diagnosis, Male), highlighting how financial factors including the long-term costs incurred over many years before having children can further complicate access to fertility services. Some AYAs also described feeling societal and cultural pressure around fertility and parenthood, with expectations about when and how they should consider family planning.

**Table:** 

Fertility and family planning (structural/contextual/mediators/processes)
Structural/contextual: policies on fertility preservation, healthcare system regulations, coverage for services
Mediators: counselling, access to information, financial support
Processes: delays in preservation, unequal access, timing conflicts with treatment
Individual AYA outcomes: delayed or missed family planning opportunities, emotional distress related to fertility uncertainty, and long-term impact on reproductive autonomy

#### Liminal survivorship and societal reintegration

After treatment, many AYAs occupy a liminal space; no longer in active care, yet not fully reintegrated into daily life, facing gaps in follow-up support, societal protections and recognition of their ongoing needs. AYAs reported that services often declined once treatment ended, with some removed from care programmes prematurely leaving them without essential long-term support even as they continued to face physical and psychological challenges: “[AYA Charity] they still had like a list of… that they care for list, and they took me off even while I was still on treatment, and when I was on maintenance. And I remember being devastated at the time because I was like, I need you more now than I needed you back then” (AYA034: 18 years at diagnosis, Male).

In some countries, AYAs encountered systemic barriers when accessing benefits and support such as housing or financial aid because they were not officially classified as disabled during the recovery period*: *“everything in the system in university and with dormitories, you are just as a healthy person. And while at the time the university had all of this policies for disabled people and to support that, I was not eligible because I did not have this disability status” (AYA038: 18 years at diagnosis, Male), demonstrating the disconnect between their health status and available societal protections.

Legal and financial protections further shaped social reintegration. In some countries, the “Right to be Forgotten”* (RTBF)* is a pivotal safeguard allowing AYAs to access mortgages or insurance without their cancer history negatively impacting them: “There’s something that I’m not dealing with right now, but I know that it can be like a future problem for me, which is like socio-economic system in [country]. It’s like a right for people after cancer treatment to be forgotten in a way, that they can have mortgage, they can do adoption after several years after the treatment. So, for now, like, it’s possible that I can’t have life insurance […] or I can’t have mortgage because they know my medical history” (AYA07: 17 years at diagnosis, Female).

**Table:** 

Liminal survivorship and societal reintegration (structural/contextual/mediators/processes)
Structural/contextual: legal protections, societal norms, policies on disability, insurance, housing
Mediators: follow-up care, long-term support, guidance for reintegration
Processes: gaps in care, barriers to benefits, identity and role reconstruction, navigating social/educational systems
Individual AYA outcomes: delayed or partial social reintegration, ongoing psychological distress, reduced access to financial or legal benefits, persistent anxiety about independence and life milestones, and a sense of being ‘in-between’ treatment and full recovery

## Discussion

Qualitative data from this cross-cultural study informed the development of a conceptual model of AYAs’ (diagnosed between the ages of 15 and 36 years) experiences of cancer and care. The model captures the multifaceted experiences of AYAs living with and beyond cancer and what matters most to them. The model highlights how experiences and outcomes are shaped by *structural/contextual* conditions (e.g. healthcare systems, policies, societal and cultural factors), *mediators* (e.g. access to information, social support, financial coverage) and lived experiences (*processes*), which together influence mental, physical, social and life course outcomes.

Compared with previous models [16], which focused on individual and healthcare-level factors with QoL as a central outcome, our model situates the AYA experience within a broader *structural/contextual* environment. This perspective acknowledges how policies, economic conditions, cultural norms and access to resources shape experiences, highlighting how disparities in care, financial burdens and social context impact the cancer trajectory [1]. This aligns with evidence showing that disparities in access and outcomes are influenced by factors such as insurance status, socioeconomic position, geographical location, sex, gender and minority status [25].

In this model, *‘*Healthcare access and navigation’ reflects the interplay between *structural/contextual* factors (healthcare system design, insurance coverage, geographical location of treatment) and *mediators* (system responsiveness, information access and social support). These shape processes including delays in care and fragmented services, influencing individual outcomes such as treatment adherence, symptom management and wellbeing. Structured patient support systems, such as the Patient Navigation Research Program, have shown that navigation services, particularly for underserved populations facing barriers to care, can improve timely diagnostic resolution and treatment initiation [26]. Integrating social prescribing within navigation programmes may further address social determinants of health by linking individuals to community resources, potentially reducing healthcare burdens and inequities [27]. Addressing these disparities requires coordinated strategies. These include policy reform (expanding ‘RTBF’ and standardising fertility preservation reimbursement), enhanced insurance coverage, patient navigation, telehealth, culturally tailored support and implementation of evidence-based AYA-specific care standards and national knowledge hubs. Together, these strategies strengthen mediators and help reduce barriers across the cancer care continuum [25, 28].

*Mediators*, including access to information, social support, financial coverage, and system responsiveness, determine how these conditions are experiences. The model highlights the importance of access to clear, timely, age-appropriate health information in helping AYAs to navigate complex healthcare systems. Gaps in information and understanding can lead to delayed decisions or missed tests. Whilst digital health literacy was not explicitly reported in the data, it is important to highlight given that young people frequently use online resources to access health information [29, 30]. Existing evidence suggests AYAs have unmet eHealth needs, which may further complicate healthcare navigation and make them vulnerable to misinformation [31]. *Mediators,* in turn, shape the *processes*, the actual experiences of AYAs. Fragmentation can occur when responsibilities for follow-up care, symptom management and patient support are unclear between primary and secondary care, leading to delays, duplicated efforts or reliance on informal networks [32]. Facilitators such as care coordinators, shared digital records, structured survivorship care plans and encouraging AYAs to actively participate in decisions (setting follow-up priorities, reporting symptoms, choosing support services) can enhance continuity, timely decision-making and engagement [32] which can directly shape AYAs’ lived experiences.

At the individual level, the model captures long-term psychological, physical and psychosocial outcomes shaped by *structural/contextual* conditions and *mediated* by access to resources and support. AYAs often have evolving support needs long after treatment ends [33]. For example, interruptions in education or employment can increase distress and negatively affect career and financial stability [1, 34]. Evidence from conditional relative survival (CRS) studies in AYAs with breast cancer show that survivors with stage I cancer reached 95% CRS from diagnosis, while those with stage II cancers reached 95% CRS 6 years post-diagnosis [35]. These findings suggest that current RTBF laws, which often require survivors to disclose their cancer history for several years, could be re-evaluated. Shortening disclosure periods could help reduce financial burdens and support social reintegration. These long-term challenges highlight the importance of comprehensive survivorship care that continues well beyond the end of treatment. An existing AYA survivorship model proposed by Gray et al. [11] emphasises the need for ongoing support across domains including care coordination, mental health, peer support, fertility, financial burden, education quality of life and late effects. Our findings identified similar themes, while further highlighting how broader structural and contextual factors, including healthcare organisation, funding, insurance policies and legal protections influence how survivorship care is experienced and accessed across different healthcare systems. Thus, our findings extend this model by demonstrating the importance of considering the wider environments (e.g. societal, cultural) and how these contexts impact on survivorship experiences and long-term outcomes.

### Implications

The qualitative findings in this study were used both to develop the conceptual model and inform a Core Outcome Set relevant to AYAs with cancer [17] which, going forward, will guide care and inform policy. Unlike traditional models, our model explicitly links structural/contextual factors, mediators and processes with individual outcomes, offering a novel framework for informing and developing patient reported outcome measures and patient reported experience measure questionnaires. Clinically, it highlights key areas for tailored support for psychosocial, navigational and structural needs of AYAs. This enables researchers to assess and prioritise the outcomes that matter most to AYAs, tailoring interventions accordingly, and strengthening understanding of the experience of AYAs with cancer.

Improving AYA outcomes requires coordinated, multi-level approaches that address system-level inequities, improve navigation and support and ensure that care is responsive to the unique needs of AYAs across diverse contexts.

### Strengths and limitations

The study draws on qualitative data from 27 AYAs and 25 HCAOPs representing a range of roles in AYA cancer and from across multiple European countries, capturing diverse nationalities, healthcare system contexts and age ranges. This multi-national perspective provides a nuanced understanding of outcomes that matter most to AYAs and forms part of the broader STRONG AYA protocol, in which AYAs with lived experience of cancer are involved in all stages of research design, implementation and dissemination.

However, it is important to acknowledge the study’s limitations. Ethnic diversity within the sample was limited, and we did not collect other equity, diversity and inclusion characteristics such as migration background, socio-economic status or disability, limiting our ability to assess representation of groups who may experience additional barriers within cancer care. Recruitment of most AYAs through a single charity, alongside the inclusion of English-speaking participants (as a first language or additional language) may have introduced selection bias. As a result, participants may have been better able to access health information than the wider AYA population, potentially limiting representation of individuals who are less connected to such organisations or whose voices are less frequently heard. Additionally, most AYAs were female and off-treatment, limiting the ability to capture the full spectrum of experiences across the cancer trajectory. The sample also did not include brain tumour survivors and had limited representation from some European healthcare systems, including France, German-speaking countries and Scandinavia. Consequently, future research including brain tumour survivors should assess whether additional emphasis is required within existing model domains, particularly the ‘Individual AYA’ outcomes domain and ‘Processes’ domain to capture experiences that may be especially relevant to this population, for example, neurocognitive sequelae, rehabilitation needs, functional independence and long-term support requirements. Furthermore, differences in healthcare organisation, survivorship care and access to specialised AYA services across European settings may require further refinement of the ‘Structural/Contextual’ domain of the model. Therefore, this study does not claim to represent the full spectrum of cross-cultural experiences across all of Europe. Future studies should test and refine the conceptual model in more diverse populations and healthcare settings, including different cultural contexts, stages of the cancer trajectory and healthcare systems that vary in the organisation of AYA services, insurance and funding arrangements, fertility preservation policies, legal protections and access to psychosocial and survivorship support. In addition, future work could also focus on whether aspects of the model are more salient/resonate more with different age sub-groups of AYAs, for example financial impact affecting older AYAs more so than the adolescents.

## Conclusion

Our study developed a novel conceptual model to reflect the multi-national perspectives and experiences of AYAs with cancer across varying European countries. By integrating perspectives from both AYAs and HCAOPs, the model highlights how *structural and contextual* factors (e.g. healthcare systems, policies, societal and cultural factors) interact with *mediators* (e.g. access to information, social support, financial coverage) to shape *processes* (e.g. delays in treatment or supportive care, fragmented care) and ultimately impact on *individual AYA outcomes*. The model supports the development of patient-centred measures and interventions that reflect priority issues for AYAs with cancer.

## Supplementary Information

Below is the link to the electronic supplementary material.

Supplementary Material 1 (DOCX 19.1 KB)

Supplementary Material 2 (DOCX 16.1 KB)

Supplementary Material 3 (DOCX 35.3 KB)

Supplementary Material 4 (DOCX 19.1 KB)

## Data Availability

The data presented in this study are available upon reasonable request from the corresponding author.
