# Supplementary material for: A conceptual model of cancer care and lived experiences in adolescents and young adults with cancer: results from the multi-national European STRONG AYA project
Source: Support Care Cancer. 2026 Sep 25;34(10):1017. doi: 10.1007/s00520-026-11170-3 (PMC13615136; doi:10.1007/s00520-026-11170-3)
Supplement: Supplementary file 2 [file 520_2026_11170_MOESM2_ESM.docx]

**Supplementary File 1. Interview guide**

**Experiences of AYAs with cancer.**

Patient interview: To start, I’d like to understand what it has been like for you to have cancer and undergo treatment.

Healthcare/allied health and other professional interview: From your point of view as a [profession]: What outcomes are especially important for adolescents and young adults with cancer.

**Outcomes of interest**

Now, I’d like to ask you about specific areas in relation to cancer outcomes. Please consider the following domains as prompts:

- Survival
- Physiological/clinical outcomes (e.g., impact on cardiac, lymphatic, or other systems)
- Physical functioning (e.g., mobility, energy levels)
- Social functioning (relationships with family, peers)
- Emotional functioning (mental health, coping, confidence in managing the disease)
- Cognitive functioning (e.g., memory, concentration)
- Overall quality of life
- Health status (e.g., general health, effects of ongoing treatment)
- Delivery of care & personal circumstances (e.g., access to care, family support)
- Economic outcomes (financial challenges, concerns about future employment)
- Societal impact (e.g., societal stigma, public awareness)
- Adverse events (e.g., side effects, complications)
- Hospital care (e.g., your experience with hospital services, communication with healthcare providers)
- Need for further intervention (e.g., additional treatments, follow-up care)
