# Supplementary material for: A conceptual model of cancer care and lived experiences in adolescents and young adults with cancer: results from the multi-national European STRONG AYA project
Source: Support Care Cancer. 2026 Sep 25;34(10):1017. doi: 10.1007/s00520-026-11170-3 (PMC13615136; doi:10.1007/s00520-026-11170-3)
Supplement: Supplementary file 3 [file 520_2026_11170_MOESM3_ESM.docx]

| **Supplementary File 2. Outcomes important to AYAs with a lived experience of cancer and associated experiences** | |
| --- | --- |
| **Perspective of Adolescent and Young Adults (AYAs) outcomes** | **Perspective of Healthcare, allied healthcare and other professionals (HCAOP) views on AYA outcomes** |
| **Healthcare and support** | |
| **Healthcare**  Experiences related to healthcare access, information, and relationships with healthcare, allied healthcare and/or other professionals (HCAOPs).   - Actively seeking information but feeling a need for more clear and relevant knowledge about their cancer and treatment - Desire to be more involved in care decisions and treatment planning - Varied satisfaction with care, depending on the level of support and communication received - Positive experiences when HCPs regularly checked in and offered emotional support - Practical help received from HCAOPs, such as arranging financial support or mobility badges - Poor support from HCAOPs, leading to feelings of neglect or feeling overlooked - Misdiagnosis resulting in delayed cancer diagnosis - Communication issues between HCPs and patients, including not being spoken to directly during appointments - Lack of trust in HCPs and feeling not taken seriously - Feeling excluded from decisions and having no control over treatment or available support - Lack of access to mental health services throughout treatment and survivorship - No signposting or clear guidance about available services and resources - Difficulties accessing medications, particularly when treatments were not available in country of residence - Limited or no spaces for friends to visit within clinical settings - Psychological support being offered only once, rather than revisited during ongoing care - Feeling that HCPs focused only on treatment, without supporting AYAs in maintaining their lives and routines outside the hospital   **Living with and beyond cancer**  Experiences after active treatment ended, including ongoing needs and adjustment.   - A strong need for ongoing psychological support after treatment ended - Wanting opportunities to speak with other people of a similar age who also had cancer - Conversations with medical teams about life implications of cancer and treatment, especially during remission - Needing physical rehabilitation to regain strength and function - Desire for nutritional guidance to support recovery - Practical challenges after treatment, such as needing help with childcare and household tasks like cooking and cleaning | **Healthcare**  Experiences related to healthcare access, information, and relationships with healthcare, allied healthcare and/or other professionals (HCAOPs).   - Feeling unheard by HCAOPs - Lack of a multidisciplinary approach involving both adult and paediatric oncology teams - Uncertainty about whether to be treated under paediatric or adult protocols   **Living with and beyond cancer**  Experiences after active treatment ended, including ongoing needs and adjustments.   - Getting back to normality in daily routines and activities - Rediscovering and maintaining relationships with family, friends, and peers - Managing ongoing symptoms at home while trying to regain independence |
| **Relationships and social functioning** | |
| **Relationships**  Experiences of navigating romantic relationships during and after cancer.   - Lowered self-esteem and body image concerns affecting the ability to form new romantic connections - Pushing partners away due to fears about mortality and the future - Difficulties engaging in online dating while undergoing treatment or managing its effects - Separation from partners during treatment - Disruption to a partner’s career plans caused by their role in caregiving - Receiving emotional and practical support from partners, including partners actively seeking information about cancer - Choosing not to pursue relationships due to the impact of cancer on themselves and on their family members - Perceptions of being less valuable because of infertility or inability to engage in intimacy - Challenges in disclosing cancer experiences to potential romantic partners - Negative perceptions of their own body and attractiveness affecting intimacy and romantic engagement - Guilt about the belief that a partner might be better off with someone “healthy” - Lack of energy making it difficult to spend time with others or maintain relationships - Dependence on parents creating barriers to romantic and social independence - Cancer helped them form more meaningful and deeper romantic relationships - Being in a relationship provided distraction and comfort during difficult times - Limited opportunities to meet new people due to being in the hospital for extended periods - Feeling that their romantic relationships were not “normal” compared to peers without cancer   **Social functioning (excl. romantic relationships)**  Experiences of friendships, peer interactions, and social life during and after cancer.   - Family members providing support by looking after young children - Supportive friends visiting in the hospital - Missing social events due to treatment or illness - Fatigue making it difficult to fully participate in school, social activities, or events such as parties - Feeling misunderstood by peers who did not fully grasp their experience - Losing friendships when peers were unable to understand or cope with the situation - Others expressing their own opinions about how the young adult should manage cancer and its effects - Receiving a lot of attention as a result of the cancer diagnosis, and feeling uncomfortable with attention - Struggling to keep up with friends’ activities due to pain or physical limitations - Becoming more selective about which friends to spend time with and who to socialize with - Socializing more with doctors and nurses on the ward than with peers - Feeling isolated because of missed opportunities for normal social development - Friendships changing significantly over the course of illness and treatment - Fatigue limiting the ability to go out or maintain social relationships - Distancing oneself from friends, either physically or emotionally - Difficulty forming new friendships or connections while in the hospital - Feeling jealous of peers who could do activities they were unable to take part in - Experiencing isolation when peers withdrew after learning about the cancer diagnosis - Guilt about burdening others by sharing true feelings and struggles - Not being invited to social events or gatherings by friends - Lacking confidence and trust when trying to form new friendships or connections   **Connecting to other peers with cancer**  Experiences of connecting with others who have cancer and are at a similar life stage.   - Feeling that connecting with others who also had cancer was important to avoid feeling alone - Difficulties connecting with others who had cancer because it served as a painful reminder of their own journey - Experiencing a sense of belonging when forming connections with peers who understood their situation - Engagement in patient advocacy, volunteering, or participating in cancer-related workshops and activities as a result of these connections   **Changes to family dynamics/roles**  Experiences of shifting roles within family and caregiving relationships.   - Changes in caregiving roles, with partners taking on more responsibilities because the young adult was unable to do certain tasks | **Relationships**  Experiences of navigating romantic relationships during and after cancer.   - Difficulties initiating or maintaining relationships due to cognitive, emotional, or physical impairments - Challenges disclosing their illness, infertility, or treatment history to new partners - Some AYAs experiencing fewer impacts on long-term relationships - Feeling that peers could not relate to issues such as being menopausal - Doubts about staying in relationships due to changes in self-perception - Partners experiencing guilt over changes in attraction or managing the emotional impact of the illness - Navigating partners’ reactions and emotions related to cancer and its effects   **Social functioning (excl. romantic relationships)**  Experiences of friendships, peer interactions, and social life during and after cancer.   - Difficulties making or maintaining friendships and changes to friendship groups - Loss of friends and feeling unable to relate to peers - Missing out on shared experiences and social activities during and after treatment - Challenges fitting back into social spaces or returning to normal routines - Avoiding social situations to reduce infection risk - Feeling the need to connect with others in a similar life stage to reduce isolation, particularly during treatment - Developing social anxiety due to long periods of isolation   **Connecting to other peers with cancer**  Experiences of connecting with others who have cancer and are at a similar life stage.   - Using platforms such as WhatsApp groups to connect with peers with similar diagnoses, increasing social support and belonging   **Changes to family dynamics/roles**  Experiences of shifting roles within family and caregiving relationships.   - Changes in caregiver roles and shifts in family dynamics - Impact of diagnosis on caring for their own children or older parents - Not being able to do previous activities or fulfil caregiving responsibilities - AYAs parents needing care themselves while coping with their own illness - Challenges in disclosing their diagnosis to young children - Managing the emotional and practical impact on children, including preparing them for changes to the parent’s health and routines |
| **Fertility and sexuality** | |
| **Fertility**  Experiences related to fertility preservation, infertility, and its emotional impact.   - Not having enough time to complete fertility preservation before treatment began - Weighing the risk to their life against the desire to have children - Feeling guilt that a partner might want children when they may not be able to have them - Facing challenges in making fertility-related decisions at a young age - Experiencing financial barriers due to the high cost of freezing eggs - Emotional and psychological distress related to infertility - Cultural or societal taboos making it difficult to discuss infertility openly - Not being given the option to preserve eggs due to needing to start treatment immediately - Having to disclose infertility to partners or potential future partners - Feelings of loss from missing the opportunity to have biological children - Gradually coming to a place of acceptance about not having biological children - Fertility preservation not being offered due to being in an adolescent age group   **Sexual intimacy**  Experiences related to intimacy and sexual relationships during and after cancer treatment.   - Difficulties engaging in sexual intimacy after treatment - Lowered self-esteem and body image concerns reducing desire for sexual intimacy - Separation from partners during treatment, preventing physical closeness or intimacy | **Fertility**  Experiences related to fertility preservation, infertility, and its emotional impact.   - Limited time to make decisions about fertility preservation due to urgent treatment schedules - Infertility affecting future relationships or marriage prospects, including within certain cultural or ethnic contexts - Sex-related disparities in fertility preservation access - Emotional impact of infertility, including distress and uncertainty about the future - Concerns about passing on cancer risk to offspring - Difficulties disclosing infertility to new or current partners - Uncertainty about what happens after fertility preservation and decisions around using stored eggs or sperm   **Sexual intimacy**  Experiences related to intimacy and sexual relationships during and after cancer treatment.   - Body image issues affecting desire for sexual intimacy - Caution around sexual activity during treatment due to increased infection risk - Limitations on ways to have penetrative sex - Vaginal dryness, low libido, and other physical changes affecting sexual intimacy - Managing partners’ emotions around challenges in the relationship or sexual intimacy - Difficulty discussing sexual intimacy with peers |
| **Identity and development** | |
| **Identity**  Experiences related to identity and loss of independence during and after cancer.   - Feeling defined by their cancer - Spending time in wards with few or no peers their age - Struggling to relate to ‘healthy’ peers - Being diagnosed during formative years, missing opportunities to attend university, start a career, or maintain a social life - Watching peers’ lives progress while their own experiences were on hold   **Loss of independence**   - Needing help with daily tasks and relying on others for support - Having to move back home and adjust to increased dependency on family | **Identity**  Experiences related to identity and loss of independence during and after cancer.   - Developing or rediscovering their sense of self during a formative period of life - Feeling a loss of identity, particularly in healthcare settings with no designated adolescent and young adult support, often being treated alongside paediatric patients - Changes in life goals and direction, including impacts on career, social life, and sexual identity - Feeling unable to relate to peers   **Loss of independence**   - Having to move back in with parents and rely on others for support - Facing risks or limitations in living independently - Experiencing lack of autonomy and inability to make the same decisions as peers   **Loss of control**   - Wanting more control over appointments and treatment decisions - Experiencing loss of opportunity to have a family, maintain relationships, or travel - Feeling unable to make the same choices as peers in daily life or long-term plans |
| **Financial impact** | |
| **Financial implications**  Experiences related to finances during and after cancer.   - Applying for or relying on financial support such as personal independent payments or reduced council tax - Struggling with the cost of childcare, such as finding a babysitter too expensive - Arranging life insurance and encountering barriers due to cancer history - Facing socioeconomic challenges, including difficulty obtaining a mortgage or other financial options because of their medical history - Paying for medication from abroad if it was unavailable in their country - Borrowing money due to inability to work or reduced income - Fundraising to cover treatment costs - Managing lower household income due to illness or time off work - Covering travel costs to and from treatment if living far from the treatment centre - Having parents or other family members take time off work to provide support or help pay for treatment and medication - No impact on finances | **Financial implications**  Experiences related to finances during and after cancer.   - Costs of preserving eggs or sperm, particularly when diagnosed at a younger age - Financial strain from being unable to work during treatment - Challenges such as not being refunded student loans or covering childcare costs - Broader socioeconomic difficulties resulting from treatment and fertility-related expenses - Considering long-term societal implications, including the ‘right to be forgotten’ regarding medical history |
| **Education and employment** | |
| **Education**  Experiences related to education during and after cancer.   - Delays in taking exams or falling behind in schoolwork - Returning to school with different peers or having to drop out entirely - Receiving little or no support from school staff - Home schooling or learning online, facing difficulties adapting to non-classroom environments - Teachers providing accommodations, such as tailored lessons or funding home schooling - Feeling isolated from peers - School sometimes serving as a distraction from their cancer   **Work**  Experiences related to work during and after cancer.   - Receiving support from employers or colleagues, including paid time off during treatment and workplace accommodations - Experiencing changes to career direction, sometimes engaging in cancer-related work as a result of their own experiences - Being unable to work due to fatigue or treatment side effects - Facing impacts on employability or career progression - Having to change career plans or not being able to pursue the career they had intended - Borrowing money due to loss of income | **Education**  Experiences related to education during and after cancer.   - Having to quit university or take extended breaks from studies - Falling behind on school or university work - Experiencing delays in future plans and goals - Impaired ability to fulfil academic or career ambitions   **Work**  Experiences related to work during and after cancer.   - Feeling pressure to return to work quickly due to sick leave entitlements - Needing to take leave or step back from their job - Experiencing delays in career plans and long-term ambitions - Facing impacts on employability and future opportunities - Managing long-term effects of treatment on work performance - Changing or redirecting career paths as a result of cancer and its treatment |
| **Physical and cognitive** | |
| **Physical**  Experiences with physical effects from cancer and its treatment.   - Hair loss - Early menopause - Weight changes, including gain or loss - Hot flushes - Night sweats - Fatigue - Muscle weakness, cramps - Reduced strength - Pain, including chronic or general pain, post-treatment chronic pain - Peripheral neuropathy - Digestive issues such as constipation, nausea, vomiting, loss of appetite, and difficulty passing wind - Mouth sores and sensitive teeth - Skin changes, including thinning - Mobility challenges - Issues with urination or incontinence - Insomnia and tinnitus   **Cognitive**  Experiences with thinking and memory during and after treatment.   - Difficulty focusing or concentrating - ‘Chemo brain’ affecting conversations and short-term memory - Struggling to do multiple tasks at once   **Behaviour**  Experiences related to strategies used to manage cancer and impact on daily life.   - Watching films or playing video games - Maintaining a positive mindset or dark sense of humour about cancer - Practicing meditation - Choosing when and how to disclose their diagnosis to feel in control - Normalising cancer by talking about it with others - Creating social media blogs - Downplaying feelings or staying positive to avoid worrying others   **Long term effects of cancer and treatment/effects post treatment**  Experiences of ongoing health issues following cancer treatment.   - Chronic pain and mobility issues, including prosthetic hips due to osteonecrosis - Diabetes - Hormonal changes - Cardiac problems - Acute pancreatitis - Pneumonia, or other post-treatment infections - Infertility and reproductive health issues - PTSD and psychological effects - Addiction to painkillers - Allergies or sensitivities to certain foods - Blood clots, anaemia, or vascular necrosis   **Managing impacts on daily routines/lives**  Experiences of adapting daily life and routines after treatment.   - Switching to online shopping to manage energy levels and health needs - Becoming more organised and planning outings carefully, such as bringing their own food or locating nearby toilets in advance | **Physical**  (listed under AYA outcomes- combined views of AYAs and HCAOPs)  **Behaviour**  Experiences related to strategies used to manage cancer and impact on daily life.   - Engaging in risky behaviours, including binge drinking - Developing alcohol dependency as a way to manage stress or emotional impact of cancer   **Long term impacts of treatment/effects post treatment**  Experiences of ongoing health issues following cancer treatment.   - Cardiac, renal, and diabetes-related complications - Cognitive difficulties affecting memory, focus, or multitasking - Osteonecrosis of hips or shoulders - Difficulty eating and managing nutrition - Peripheral neuropathy and related physical limitations - Social challenges, including difficulty forming friendships due to long periods of isolation - Social anxiety and feeling different from peers, making it harder to engage socially |
| **Psychosocial and emotional** | |
| **Mental health/psychological**  Experiences related to mental health and psychological wellbeing during and after cancer treatment.   - Fear of recurrence and fear of death - Anxiety, panic attacks, and stress about the future - Depression, sadness, and feeling overwhelmed - Shock, suicidal thoughts, and feeling betrayed by their own body - Difficulty doing things or being the person they were before cancer - Isolating themselves or avoiding peers and emotions - Survivors’ guilt and social anxiety from long periods of isolation - Low self-esteem and hiding feelings to avoid worrying others - Feeling stuck while peers progressed in their lives - Facing adult decisions about life and death at a young age - Difficulties reintegrating into society, including challenges in career and family planning - Experiencing things peers their age had not   **Positive psychological function**   - Acceptance of cancer and its effects - Feeling in control of their life rather than letting cancer dictate it - Finding benefits in their experience, such as broader perspective, enjoying life, and focusing on what matters   **Body image**  Experienced related to body image.   - Lowered self-esteem, lack of confidence, and feeling disconnected from their body - Feeling self-conscious or betrayed by side effects such as hair loss, loss of eyebrows, or weight changes - Struggling with wigs, hot weather, or parents’ reactions to body changes - Overcompensating through fitness or developing eating disorders - Feeling unrecognisable or “alien-like” due to body changes - Hair loss affecting identity and social interactions - Feeling confident or empowered despite changes, including baldness - Using humour to cope with body changes - Enjoying wigs or preferring short hair as a new style | **Mental health/psychological**  Experiences related to mental health and psychological wellbeing during and after cancer treatment.   - Health anxiety and fear of relapse or dying - Pre-existing mental health conditions influencing their experience - Impact of body image and loss of control affect mental wellbeing - Worries and anxieties about the future - Depression, isolation, and feeling different from peers - Grieving the life they had before cancer and managing expectations versus reality - Engaging in risk-taking behaviours or struggling with social participation due to feeling different - Survivors’ guilt and processing long-term psychological effects, including being triggered by reminders - High need for mental health support and awareness of elevated suicide risk in this population - Reflecting on life satisfaction and making sense of their experience - Worrying about survival and thinking more about death than existence   **Body image**  Experienced related to body image.   - Changes in appearance, including hair loss, scarring from PICC lines, and weight gain from steroids - Impacts on self-esteem and confidence in their body - Feeling their body had let them down - Challenges in forming new romantic or social connections due to appearance or self-consciousness |
