# Supplementary material for: A conceptual model of cancer care and lived experiences in adolescents and young adults with cancer: results from the multi-national European STRONG AYA project
Source: Support Care Cancer. 2026 Sep 25;34(10):1017. doi: 10.1007/s00520-026-11170-3 (PMC13615136; doi:10.1007/s00520-026-11170-3)
Supplement: Supplementary file 4 [file 520_2026_11170_MOESM4_ESM.docx]

**Supplementary file 3. Mapping of interview findings to the conceptual model**

| **ID** | **Code** | **Quote** | **Considerations**  **(e.g. culturally/societally/structurally)** |
| --- | --- | --- | --- |
| STRONG AYA 032 | Independently sourcing medication | *“Because the medicine is also sometimes hard to find ... And well, sometimes you have to get it from abroad because sometimes it might not just be available in the country. “* | Participant had to source medication from abroad (in this example, it is chemotherapy) independently, rather than through the healthcare system.  Structural / Contextual: Geography + regional inequality; healthcare system design (availability of chemotherapy in country).  Processes: Delayed or disrupted decision-making; fragmented transitions (participant must independently source treatment).  Outcome domain: Healthcare access and navigation. |
| STRONG AYA 007 | Legal and societal barriers to post-treatment life | *“There’s something that I’m not dealing with right now, but I know that it can be like a future problem for me, which is like socio-economic system in [country], which is not really supportive. There is no talking about right to be forgotten, if you heard about it. It’s like a right for people after cancer treatment to be forgotten in a way….So for now, like, it’s possible that I can’t have life insurance because they know my medical history or I can’t have mortgage because they know my medical history”* | Country has less support for individual rights after treatment. Survivors therefore have more challenges reintegrating into society as they struggle to get health insurance or a mortgage.  Structural / Contextual: Legal protections (*Right to be Forgotten*) and societal treatment of cancer survivors.  Processes: Cumulative challenges in navigating care; barriers to reintegration into society.  Outcome domain: Liminal survivorship and challenges returning to society; Economic instability and hardship. |
| STRONG AYA 035 | Access to urgent care and financial barriers | *“I just found some place where I could do the CT in [country] in the health system is like I can, like, I can try to get the CT for free because I have the taxes and then have insurance. But of course it takes some times and she told me that this is very urgent and I should do as far as as soon as possible. And thankfully, my family and I, we’ve had resources to do this just on the next day. And of course, like to pay for it for such like I would say it’s quite amount of money…”* | Challenges in accessing urgent healthcare where the participant navigates delays in the public system despite having insurance, highlighting the financial burden of accessing care immediately.  Structural / Contextual: Healthcare system design; insurance rules; public/private healthcare.  Mediators: Financial coverage; system responsiveness; social support availability (family support).  Processes: Social and family support; delayed or disrupted decision-making.  Outcome domains: Healthcare access and navigation; Economic instability and hardship. |
| STRONG HCP 002 | Masculinity norms as barriers to mental health help-seeking | *“The patients I worry about are the particularly young men who sort of bluster their way through treatment and have this I'll be fine approach. Nothing's going to bother me and or they see it as a weakness to acknowledge that they're very sad. And then almost inevitably you know at some moment of stress they have some sort of major depressive episode or anxiety episode or something….”* | Societal expectations around masculinity may influence young men’s willingness to acknowledge emotional distress during cancer treatment. It highlights the stigma surrounding mental health, particularly for men.  Structural / Contextual: Cultural and societal norms around masculinity and mental health (masculinity, shame).  Processes: Avoidance of help-seeking.  Mediators: Availability of social support; mental health services (accessed variably depending on willingness).  Individual outcomes: Mental health and emotional strain. |
| STRONG HCP 021 | Geographical barriers to care | *“[…] And we do have other psychologists in the hospital, but it's difficult because we're a centre which can take three to four hours to drive to. The kind of treatment service and input that they need is difficult to achieve if they're not coming up to the hospital every week.”* | Challenge of accessing mental health support due to geographic distance, which can be a barrier to consistent care for patients in rural or remote areas. Importance of ensuring that patients in distant locations can receive the same level of psychological support without the logistical and financial burden of frequent travel.  Structural / Contextual: Geography + regional inequality; healthcare system design.  Mediators: System responsiveness; access to mental health support.  Processes: Delayed or disrupted access; cumulative challenges in navigating care.  Outcome domains: Healthcare access and navigation.  Individual outcomes: Mental health and emotional strain. |
| STRONG HCP 008 | Inequality in fertility preservation access | *“The inequalities around fertility offers, just in general... I think if you're a male, you get a better offer than you do a female because the time it takes to freeze your eggs. So it depends who your clinician is, it depends what you get offered. If you are a male, you tend to get offered sperm banking because it's a very quick process, it's literally you can book you in for the same day or the next day…Whereas if you're a female, it can take between four and eight weeks to harvest your eggs.”* | Disparities in fertility preservation options for young cancer patients. It emphasises the unequal access to fertility services, with male patients often receiving quicker and more immediate options (sperm banking), while female patients face longer, more complex processes for egg harvesting.  Structural / Contextual: Healthcare system design (sex-differentiated fertility pathways).  Mediators: System responsiveness (timely and coordinated fertility care); access to fertility information.  Processes: Gatekeeping and unequal access (clinician discretion, timing differences, referral pathways); delayed or disrupted decision-making (conflict between treatment start and fertility preservation timelines).  Outcome domains: Fertility inequities and family-planning barriers.  Individual outcomes: Fertility outcomes and autonomy in reproductive decision-making. |
